# Serum soluble CD40 is associated with liver injury in patients with chronic hepatitis B

**DOI:** 10.3892/etm.2015.2182

**Published:** 2015-01-14

**Authors:** HONG-HUI SHEN, BING-KE BAI, YA-QING WANG, GUANG-DE ZHOU, JUN HOU, YAN HU, JING-MIN ZHAO, BAO-SEN LI, HAI-LI HUANG, PAN-YONG MAO

**Affiliations:** 1 Institute of Infectious Diseases, Beijing 302 Hospital, Beijing 100039, P.R. China; 2 Department of Gastroenterology, Beijing 305 Hospital, Beijing 100017, P.R. China; 3 Department of Pathology, Beijing 302 Hospital, Beijing 100039, P.R. China; 4 Department of Gastroenterology, General Hospital of PLA, Beijing 100853, P.R. China

**Keywords:** fibrosis, hepatocyte, immune, necroinflammatory

## Abstract

Soluble cluster of differentiation 40 (sCD40) is proteolytically cleaved from membrane-bound CD40 and binds to CD154, thereby inhibiting CD40-CD154-mediated immune responses. The aim of the present study was to clarify the role of sCD40 in chronic hepatitis B (CHB). The sCD40 levels in sera from 132 patients with CHB and 33 healthy individuals were retrospectively measured. sCD40 concentrations in patients with CHB were higher than those in healthy controls, and sCD40 levels correlated positively with serum levels of the liver dysfunction biomarkers alanine transaminase (ALT) and aspartate transaminase (AST). sCD40 concentrations increased with a rise in the severity of liver necroinflammation and fibrosis. Patients with >75% liver tissue staining positive for hepatitis B virus (HBV) antigen expression showed significantly lower sCD40 levels than those who stained negative for the HBV antigen. The area under the receiver operating characteristic curve of sCD40 was greater than that of ALT and AST; thus, sCD40 levels have a high diagnostic accuracy for detecting severe liver inflammation in patients with CHB, and could serve as an immunological marker of hepatic tissue injury.

## Introduction

Cluster of differentiation (CD) 40 is a member of the tumor necrosis factor (TNF) receptor superfamily. It is expressed on the surface of immune cells, including B cells, monocytes, macrophages, dendritic cells (DCs) and activated T cells, as well as on the surface of non-immune cells, such as epithelial, endothelial and mesenchymal cells (including fibroblasts, myofibroblasts, synoviocytes and stellate cells) (1,2).

CD154, a CD40 ligand, is preferentially expressed by activated T cells, activated DCs and activated platelets, although it can also be variably expressed by monocytes and mononuclear phagocytes, as well as natural killer, B, CD8^+^ T, human vascular endothelial and smooth muscle cells (1–4). The CD40-CD154 interaction plays a critical role in the regulation of humoral immunity, cell-mediated immunity and inflammation, and results in the production of numerous chemokines and cytokines, the upregulation of adhesion molecules, the secretion of matrix metalloproteinases (MMPs) and the induction of apoptosis (3). An impaired CD40-CD154 interaction leads to humoral and cellular immunodeficiency; thus, the CD40-CD154 co-stimulatory pathway is associated with the pathogenesis of several diseases, including autoimmune thyroiditis, type 1 diabetes, inflammatory bowel disease, psoriasis, multiple sclerosis, rheumatoid arthritis and systemic lupus erythematosus (4,5).

Soluble CD40 (sCD40) comprises the extracellular domain of CD40 and is generated via proteolytic cleavage from the surface of CD40-expressing cells (6,7). The binding of CD40 to its receptor on CD40-expressing cells can lead to enhanced sCD40 release (7). sCD40, as a CD40 antagonist, is able to bind to CD154 and inhibit CD40-CD154-mediated immune responses by blocking the interaction of CD40 itself with CD154 (8–10). Circulating sCD40 levels are elevated in patients with chronic renal failure, chronic liver diseases, Alzheimer’s disease, systemic sclerosis and hematological malignancies (10–14).

The CD40-CD154 co-stimulatory pathway is associated with liver injury and hepatocyte apoptosis (15–17). Kupffer cells and hepatocytes can express elevated levels of CD40 in hepatitis C virus-associated chronic liver disease (18,19). CD40-activated B cells and macrophages produce inflammatory cytokines and contribute to the pathogenesis of necroinflammatory liver disease (20). Although CD40-expressing cells have been studied extensively in patients with liver diseases, limited information is available regarding the serum levels of sCD40 in these conditions. Schmilovitz-Weiss *et al* (14) reported that sCD40 levels were significantly higher in patients with liver disease than those in controls; however, in their study, only a few patients with chronic hepatitis B (CHB) were enrolled, and these patients were not analyzed as a separate group.

Since the pathogenesis in different liver diseases varies, and the role of sCD40 in CHB has not been clarified, the levels of sCD40 in sera from patients with CHB were retrospectively measured in the present study, and their association with biochemical abnormalities and liver histological characteristics were analyzed in detail.

## Materials and methods

### Ethics statement

The present study was approved by the Ethics Committee of Beijing 302 Hospital (Beijing, China), and written informed consent was obtained from each subject.

### Subjects

The patients enrolled in this study had been admitted to Beijing 302 Hospital between December 2001 and December 2005. The diagnoses were based on standard clinical, biochemical and histological criteria, according to the guidelines for CHB (21). Patients with CHB had been hepatitis B virus surface antigen (HBsAg)-positive for at least six months and exhibited symptoms of viral hepatitis and abnormal hepatic function during this period. All the patients were infected solely with hepatitis B virus (HBV), and no other cause of liver disease (such as other virus infections, autoimmune disease, drug hypersensitivity, significant alcohol intake, hemochromatosis or Wilson’s disease) had been diagnosed in any of the patients. Patients were excluded from this study if they had other diseases, such as heart disease, nephritis, cholecystitis and gastritis, or if they had received antiviral or immunomodulatory treatment. Blood samples from healthy donors that had reported to the hospital for physical examination in the corresponding period were used as controls.

### Detection of sCD40

At the time of admission, sera from the healthy individuals and patients with CHB were collected and stored at −70°C. In the present study, serum sCD40 concentrations were simultaneously measured using an ELISA according to the protocol for the sCD40 Module Set (Bender MedSystems GmbH, Vienna, Austria).

### Laboratory data

Data on laboratory indices, including the serum levels of alanine transaminase (ALT), aspartate transaminase (AST), total bilirubin, direct bilirubin (Dbil), globulin, cholinesterase, alkaline phosphatase (ALP), γ-glutamyl transpeptidase (γGT), total bile acids (TBA) and hepatitis B virus extracellular antigen (HBeAg), which had been measured on the date of serum collection, were retrospectively obtained from the hospital records.

### Histology and immunohistochemistry

The patients who had CHB and whose data were included in the study had undergone a liver biopsy with a Menghini needle within one week of the date of serum collection. These liver biopsy specimens were evaluated by a hepatic pathologist who was unaware of the patients’ clinical and biochemical data or sCD40 levels. The specimens were graded according to the modified histological activity index (HAI) scoring system described by Ishak *et al* (22). The modified HAI grading and staging scores provided a semi-quantitative assessment of the observed histological features. The grading described the intensity of necroinflammatory activity, while the staging denoted the degree of fibrosis and architectural changes that occurred in chronic hepatitis (22).

The expression of HBsAg and HBV core antigen (HBcAg) was determined in formalin-fixed, paraffin-embedded tissue specimens by indirect immunoperoxidase staining, with semi-quantitative scoring (0, negative; 1, <25%; 2, 25–49%; 3, 50–74% and 4, ≥75%). Briefly, the liver tissue (5 μm) was incubated with mouse anti-human HBsAg or HBcAg antibodies (MS-314 and RB-1413, respectively; 1:50; Maixin Biotech; Fuzhou, China) overnight at 4°C following the blocking of endogenous peroxidase activity with 0.3% H_2_O_2_. 3,3′-diaminobenzidine was used as the substrate followed by counterstaining with hematoxylin for single staining.

### Statistical analysis

Statistical analyses were performed using SPSS software (version 12.0; SPSS Inc., Chicago, IL, USA). Quantitative variables were statistically tested for normality of distribution. Normal quantitative variables are presented as the mean ± standard deviation and were analyzed using parametric tests. The values of serum sCD40 concentration were transformed to their natural log values and analyzed by one-way analysis of variance and the Student’s t-test. Skewed quantitative variables are expressed as the median and interquartile range (IQR) and analyzed using the Kruskal-Wallis or Mann-Whitney tests. Associations between sCD40 concentrations and other variables were tested using Spearman’s rank correlation coefficient. χ^2^ or Fisher’s exact tests were used for categorical variables. Multiple regression and comparison of the areas under the receiver operating characteristic (ROC) curves for sCD40, ALT and AST were performed using MedCalc software (version 12.0.4; MedCalc Corp., Mariakerke, Belgium). P<0.05 was considered to indicate a statistically significant difference.

## Results

### Patient characteristics

sCD40 concentrations were measured in 132 patients with CHB and 33 healthy individuals, with median ages of 21.7 years (IQR, 15.3 years) and 21.8 years (IQR, 5.3 years), respectively. No significant differences in age were observed between the patients with CHB and healthy individuals (P=0.157). The association between serum sCD40 concentration and age was insignificant, with a correlation coefficient of −0.090 for patients with CHB (P=0.306) and −0.005 for healthy individuals (P=0.979). In addition, the proportion of male subjects was similar in the two groups (77.3 vs. 87.9%; Pearson χ^2^ value, 2.266; P=0.312).

### Correlations between sCD40 concentration and laboratory indices

The laboratory data of patients with CHB were correlated with serum sCD40 levels using Spearman’s rank correlation coefficient (Table I). The sCD40 levels in patients with CHB correlated positively with serum levels of ALT, AST, Dbil, globulin, ALP, γGT and TBA.

### sCD40 concentration is elevated with aggravated liver injury in patients with CHB

The sCD40 concentrations in patients with CHB are shown in Table II. sCD40 levels in patients with CHB were higher than those in healthy controls (P<0.001). The difference in sCD40 concentrations between serum HBeAg-positive and HBeAg-negative CHB patients was not significant (P=0.488). sCD40 concentrations in patients with CHB correlated positively with the Ishak score (Spearman correlation coefficient, 0.506; P<0.001).

**Figure 1:**
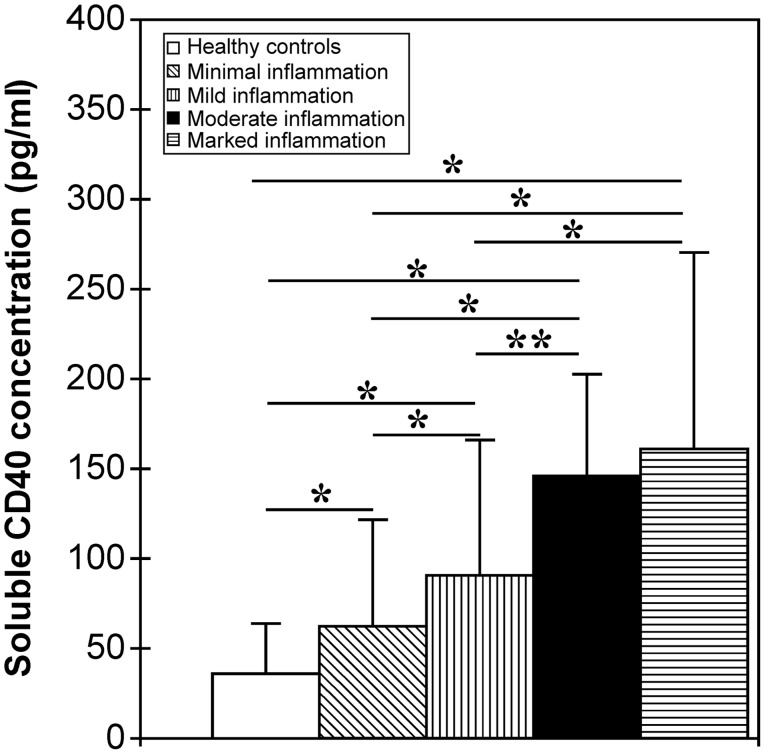
Soluble cluster of differentiation 40 (sCD40) levels in patients with different degrees of liver inflammation. The serum sCD40 concentrations were natural log-transformed and analyzed by one-way analysis of variance. ^*^P<0.01, ^**^P<0.05.

To investigate the sCD40 levels in patients with different intensities of liver inflammation, the patients with CHB were distributed into four groups based on their Ishak scores: Minimal inflammation, scores 1–4; mild inflammation, scores 5–8; moderate inflammation, scores 9–12; and marked inflammation, scores 13–18. The correlation between these groups and the sCD40 levels was then assessed. It was found that sCD40 concentrations gradually rose with increasing liver necroinflammation. The liver-inflammation groups showed a significantly higher sCD40 concentration than did the healthy control group (P<0.001, Fig. 1). The sCD40 concentration in patients with CHB with minimal inflammation was significantly lower than that in patients with mild, moderate and marked inflammation (P<0.01), and the sCD40 concentration in patients with CHB with mild inflammation was significantly lower than that in patients with moderate and marked inflammation (P<0.05, Fig. 1). The difference in sCD40 concentrations, however, between individuals with moderate inflammation and those with marked inflammation was not significant (P=0.186, Fig. 1).

**Figure 2:**
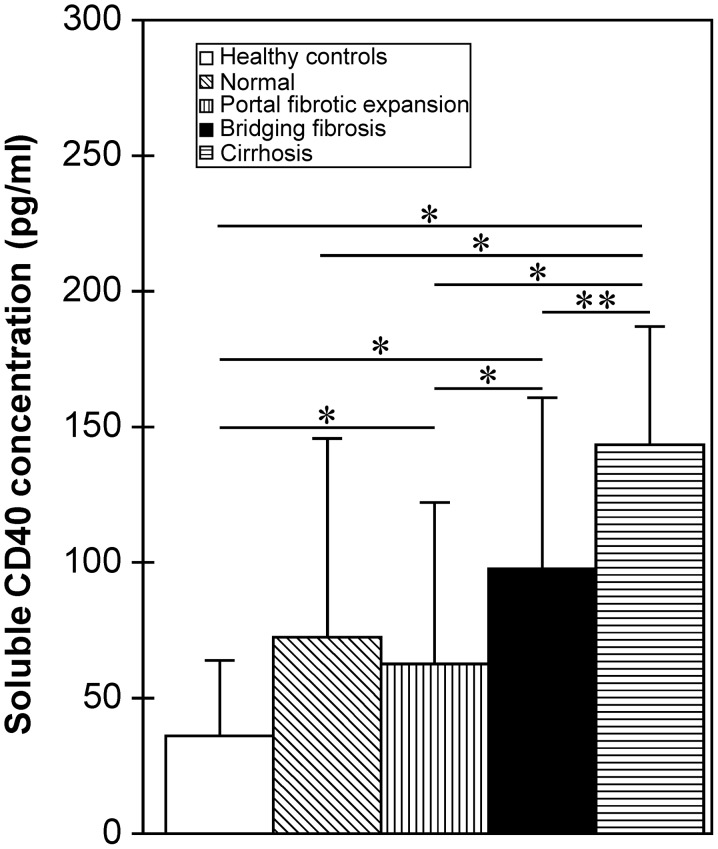
Soluble cluster of differentiation 40 (sCD40) levels in patients with different degrees of liver fibrosis. The serum sCD40 concentrations were natural log-transformed and analyzed by one-way analysis of variance. ^*^P<0.01, ^**^P<0.05.

The sCD40 concentration in patients with CHB also positively correlated with the Ishak fibrosis staging score (Spearman correlation coefficient, 0.395; P<0.001). Patients with CHB with different fibrosis staging scores were distributed into four groups: normal, score 0; portal fibrotic expansion, scores 1–2; bridging fibrosis, scores 3–4; and cirrhosis, scores 5–6. The correlation between sCD40 level and these groups was then investigated. It was found that the sCD40 concentration gradually increased with the aggravation of liver fibrosis (Table II). The difference in sCD40 levels between patients with CHB without fibrosis (normal group) and the healthy controls was not significant (P=0.072), whereas groups with portal fibrotic expansion, bridging fibrosis and cirrhosis showed significantly higher sCD40 concentrations than did healthy controls (P<0.001, Fig. 2). The sCD40 concentration in patients with CHB with portal fibrotic expansion was significantly lower than that in patients with bridging fibrosis or cirrhosis (P<0.01), and the sCD40 concentration in patients with CHB with cirrhosis was significantly higher than that in patients with bridging fibrosis (P<0.05; Fig. 2).

### sCD40 concentration is reduced with an increase in hepatic HBV antigen expression

**Figure 3:**
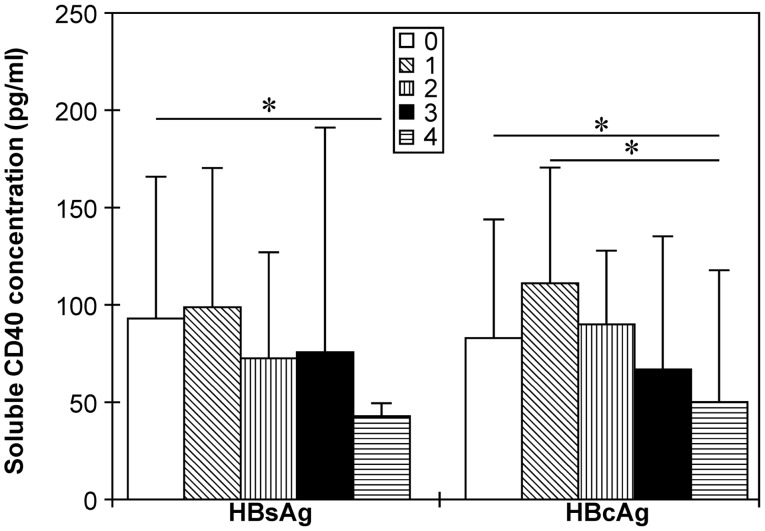
Soluble cluster of differentiation 40 (sCD40) concentrations in patients with chronic hepatitis B with different intensities of hepatitis B virus (HBV) antigen expression in liver tissue. Serum sCD40 concentrations were natural log-transformed and analyzed by one-way analysis of variance. Hepatic HBV antigen expression was analyzed using a semi-quantitative indirect immunoperoxidase staining method (0, negative; 1, ≤25%; 2, 25–50%; 3, 50–75%; 4, ≥75%). ^*^P<0.05.

sCD40 concentration correlated negatively with HBsAg (r=−0.194; P=0.053) and HBcAg (r=−0.212; P<0.05) expression in the liver. The sCD40 concentration in patients with HBsAg expression present in >75% of liver tissue was significantly lower than that in patients without detectable HBsAg expression. Furthermore, sCD40 levels in patients with HBcAg expression in >75% of liver tissue were significantly lower than those in patients with HBcAg expression in <25% of liver tissue (Fig. 3).

### sCD40 concentration has high a diagnostic accuracy for detecting severe liver injury in patients with CHB

To further investigate the diagnostic value of sCD40 levels in liver injury, ROC analysis was performed (Table III). The area under the curve (AUC) of sCD40 for discriminating patients with CHB from healthy individuals was 0.843 (P<0.001), with a sensitivity, specificity, positive-predictive value and negative-predictive value (NPV) of 0.712, 0.848, 0.949 and 0.424, respectively. The AUC of sCD40 for diagnosing patients with CHB with moderate and marked inflammation (necroinflammatory grading score >9), patients with marked inflammation (necroinflammatory grading score >13) and patients with cirrhosis (fibrosis staging score >5) was 0.820, 0.855 and 0.783, respectively, with a high sensitivity, specificity and NPV (Table III).

**Figure 4:**
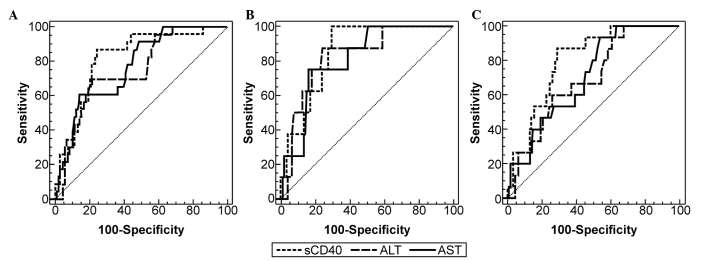
Comparison of serum sCD40, ALT and AST ROC curves for the detection of different degrees of liver injury. (A) Diagnosis of patients with CHB with moderate and marked inflammation (necroinflammatory grading score ≥9). (B) Diagnosis of patients with CHB with marked inflammation (necroinflammatory grading score ≥13). (C) Diagnosis of patients with CHB and cirrhosis (fibrosis staging score ≥5). All P-values >0.05, when the area under the ROC curve of sCD40 was compared with that of ALT or AST. sCD40, soluble cluster of differentiation 40; ALT, alanine transaminase; AST, aspartate transaminase; ROC, receiver operating characteristic; CHB, chronic hepatitis B.

Comparisons of ROC curves of serum sCD40, ALT and AST to detect different degrees of liver injury in patients with CHB are shown in Fig. 4. Although the differences between the AUC of sCD40 and ALT or sCD40 and AST were not statistically significant, the AUC of sCD40 was greater than the AUC of ALT and AST, when used to diagnose patients with CHB with moderate and marked inflammation (0.817 vs. 0.752 and 0.769, respectively), patients with marked inflammation (0.852 vs. 0.829 and 0.816, respectively) and patients with cirrhosis (0.799 vs. 0.694 and 0.709, respectively). This indicated that sCD40 may have a slightly higher diagnostic accuracy than ALT and AST for detecting severe liver injury in patients with CHB.

The necroinflammatory grading score, fibrosis staging score and levels of sCD40, ALT, and AST were introduced as variables into a stepwise multiple regression analysis. The regression equation used was as follows: necroinflammatory grading score = −0.408 + 2.031 × fibrosis-staging score + 0.008 × sCD40 level. The finding that the ALT and AST variables were excluded from the regression equation further supported our finding of a stronger association between liver inflammation and sCD40 level than between liver inflammation and ALT and AST levels.

## Discussion

Hepatitis B is the most common chronic liver disease in China. The CD40-CD154 co-stimulatory pathway is involved in the pathogenesis of CHB (16). Intrahepatic CD40 expression has been shown to be upregulated on the surface of hepatocytes in CHB and to cause liver injury (23,24). Furthermore, activation of CD40 on hepatocytes and cholangiocytes is critical for amplifying Fas-mediated apoptosis in the human liver (25). The CD40 molecules on the cell surface that are activated by CD154 can trigger sCD40 release (12,13), and it is known that an increased expression of CD40 on the membrane is associated with abundant release of its soluble form (6). The elevated serum levels of sCD40 in patients with CHB observed in the present study may therefore be the result of increased shedding of this peptide from CD40-expressing cells and decreased elimination by the impaired liver.

Serum ALT, AST, Dbil, ALP, γGT and TBA are markers of liver dysfunction and are associated with liver injury. Positive associations between sCD40 levels and these biochemical indices, as well as liver necroinflammatory grading scores, in patients with CHB observed in the present study suggested an involvement of sCD40 in liver inflammation. The observation that the already-elevated sCD40 concentration in patients with CHB gradually increased with increasing severity of liver necroinflammation or fibrosis also supported this finding.

CD40-CD154 interactions in the liver can induce immune responses, inflammatory injury and hepatocyte apoptosis (26,27). The elevated levels of sCD40 in CHB can compete with membrane CD40 for binding to CD154, thereby inhibiting CD40-CD154 interactions and ultimately achieving effective negative feedback control of the CD40-CD154-mediated immune response and hepatocyte apoptosis (7,10,12,13,28). Thus, we speculated that inhibition of the immune responses mediated by sCD40 shedding would prevent liver tissue from excessive injury (10,13). ROC and multiple regression analysis of sCD40 showed that sCD40 levels have higher diagnostic accuracy than do those of ALT and AST when used to detect severe liver inflammation in patients with CHB. This suggested that serum sCD40 levels could serve as a novel immunological marker of hepatic tissue injury in such patients.

Liver fibrosis represents a pathological accumulation of extracellular matrix (ECM) components, which are mainly degraded by the MMPs, e.g. MMP-1, MMP-2, MMP-3 and MMP-9 (29–31). CD40 ligation on monocytes/macrophages and endothelial cells by CD154 can increase the release of MMP-1, MMP-3, MMP-9 and activated MMP-2 (32,33). Low activity of MMPs may contribute to the excess deposition of intrahepatic ECM and may thus play an important role in the process of liver fibrosis. Previously, it was found that the serum levels of MMP-1, MMP-2 and MMP-9 in patients with CHB were significantly lower than those in healthy controls, and serum MMP-1 levels negatively correlated with fibrosis stage and inflammation grade (34–37). Although the tissue inhibitors of MMP-1 and -2 are considered to be the major reasons for inhibition of MMP activity, sCD40 may also reduce MMP expression by blocking the CD40-CD154 interaction. This could reduce hepatic degradation of ECM and result in liver fibrosis. This hypothesis is also supported by the positive correlation between serum sCD40 levels and hepatic fibrosis observed in the present study (38); however, the mechanism underlying the role played by sCD40 in liver fibrosis requires further clarification.

The CD40-CD154 interaction represents a critical co-stimulatory pathway that modulates the immune response. CD40 binding to intrahepatic antigen-presenting cells has been shown to induce the secretion of antiviral cytokines, such as interleukin-12 and TNF-α, and then to inhibit HBV replication in the liver of HBV-transgenic mice (16). sCD40 can inhibit the production of antiviral cytokines and may therefore weaken the CD40-CD154-mediated antiviral immune response (39). The results from the present study, however, suggest that the elevation of serum sCD40 levels in patients with CHB is associated with downregulation of intrahepatic HBV antigen expression. The mechanism by which sCD40 elevation inhibits intrahepatic HBV antigen expression is unknown. It is possible that a CD40-CD154-mediated antiviral immune response contributes to both the inhibition of HBV antigen expression and the shedding of sCD40. These two consequences have no direct association, as patients with serum HBeAg-positive and HBeAg-negative CHB showed similar sCD40 levels.

In conclusion, the present results suggest that sCD40 plays an important role in the pathogenesis of CHB. sCD40 may serve as a diagnostic and immunological marker of liver injury and can act as a negative regulator of the CD40-CD154 interaction in patients with CHB.

## Figures and Tables

**Table I:** Correlations between soluble cluster of differentiation 40 concentration and laboratory indices in patients with chronic hepatitis B.

Variables	N	Value^a^	Correlation coefficient	P-value
ALT (U/l)	130	59 (101)^a^	0.487	<0.001
AST (U/l)	130	45 (66)^a^	0.492	<0.001
Total bilirubin (μmol/l)	130	10.2 (6.6)^a^	0.170	0.053
Direct bilirubin (μmol/l)	130	2.4 (3.6)^a^	0.226	0.010
Globulin (g/l)	130	24.9 (3.9)^b^	0.239	0.006
Cholinesterase (U/l)	122	8321 (2453)^b^	−0.131	0.150
ALP (U/l)	122	96 (156)^a^	0.232	0.010
γGT (U/l)	122	29 (34)^a^	0.499	<0.001
TBA (μmol/l)	122	7 (6)^a^	0.327	<0.001

a Values showed a skewed distribution and are presented as the median (interquartile range);

b values demonstrated a normal distribution and are presented as the mean (standard deviation).

ALT, alanine transaminase; AST, aspartate transaminase; ALP, alkaline phosphatase; γGT, γ-glutamyl transpeptidase; TBA, total bile acids.

**Table II:** Soluble cluster of differentiation 40 concentrations in patients with chronic hepatitis B.

Grouping	N	Geometric mean (pg/ml)
Chronic hepatitis B	132	82.8
HBeAg-positive	87	78.7
HBeAg-negative	45	92.2
Necroinflammatory grading score^a^		
0–4	66	61.8
5–8	43	91.7
9–12	15	139.0
13–18	8	203.2
Fibrosis staging score^a^		
0	5	59.0
1–2	67	66.1
3–4	44	96.2
5–6	16	157.2
Healthy controls	33	32.8

a Patients with chronic hepatitis B were grouped according to the Ishak score (22).

**Table III:** Diagnostic accuracy of soluble cluster of differentiation 40 for the detection of different degrees of liver injury in patients with CHB.

Detection subject	AUC	P-value	Cut-off value (pg/ml)	Sensitivity	Specificity	PPV	NPV
CHB patients	0.843	<0.001	57.5	0.712	0.848	0.949	0.424
CHB patients with grading score ≥5^a^	0.737	<0.001	103.3	0.606	0.818	0.769	0.675
CHB patients with grading score ≥9^a^	0.820	<0.001	116.7	0.870	0.761	0.435	0.965
CHB patients with grading score ≥13^a^	0.855	<0.001	118.4	1.000	0.710	0.182	1.000
CHB patients with staging score ≥3^b^	0.705	<0.001	103.3	0.583	0.764	0.673	0.688
CHB patients with staging score ≥5^b^	0.783	<0.001	116.7	0.812	0.716	0.283	0.965

a Necroinflammatory grading score;

b fibrosis staging score;

AUC, area under the receiver operating characteristic curve; PPV, positive-predictive value; NPV, negative-predictive value; CHB, chronic hepatitis B.
